# Perspective-Taking: In Search of a Theory

**DOI:** 10.3390/vision4020030

**Published:** 2020-06-01

**Authors:** Geoff G. Cole, Abbie C. Millett, Steven Samuel, Madeline J. Eacott

**Affiliations:** 1 Centre for Brain Science, University of Essex, Colchester CO4 3SQ, UK; ssamuea@essex.ac.uk (S.S.); m.j.eacott@essex.ac.uk (M.J.E.); 2 School of Social Sciences and Humanities, University of Suffolk, Ipswich IP4 1QJ, UK; a.millett2@uos.ac.uk

**Keywords:** perspective-taking, social attention, mental imagery, vision, gaze cueing

## Abstract

Perspective-taking has been one of the central concerns of work on social attention and developmental psychology for the past 60 years. Despite its prominence, there is no formal description of what it means to represent another’s viewpoint. The present article argues that such a description is now required in the form of theory—a theory that should address a number of issues that are central to the notion of assuming another’s viewpoint. After suggesting that the mental imagery debate provides a good framework for understanding some of the issues and problems surrounding perspective-taking, we set out nine points that we believe any theory of perspective-taking should consider.

## 1. Introduction

**Figure 1:**
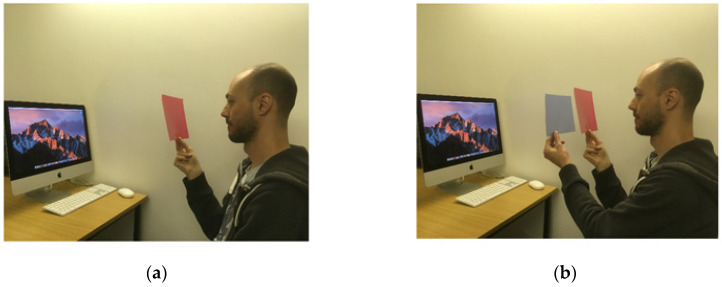
A test of visual perspective taking (**a**,**b**).

Figure 1a shows an agent viewing an image through a red filter. Take his perspective and ask yourself what is the dominant colour the image appears to the agent. If your answer is “red”, do you think you arrived at this (correct) answer because you were able to take his *visual perspective*? In other words, can you genuinely represent what a scene looks like from the position of another person? If you believe so, consider Figure 1b. Again, take the perspective of the agent, this time holding and looking through two overlapping filters simultaneously, a red filter and a blue filter. What colour is the scene now tinged in? The fact that the vast majority of people cannot derive the correct answer suggests that cognitive scientists need to be clearer in the way visual perspective-taking is conceived. In the present article, we will argue that the notion of perspective-taking is vague in the sense that a description of what it means to assume another’s viewpoint has not been forthcoming. Theory is now required. 

## 2. Perspective-Taking

In 1956, Piaget and Inhelder [1] published a seminal article in which they examined the developing child’s ability to assume a visual perspective different to its own. In the famous three mountains task, children of various ages were asked to judge what another agent could see from their viewpoint. This initiated an abundance of work assessing, for example, how perspective-taking relates to broader developmental stages [2] and how familiarity with the display items modulates responses [3]. The visual perspective-taking notion has also been applied to many non-developmental areas, such as ‘joint action’ in which two participants sit adjacently and share responses (i.e., take turns) on a target discrimination task (for a review, see [4]). While early explanations of the kind of effects that occur in this paradigm included the idea that another’s actions become incorporated within an observer’s own motor representations (e.g., [5]), more recently it has been argued that joint action effects can now be explained with a form of perspective-taking [6].

The notion of perspective-taking has, however, suffered from a lack of clarity of the process (es) under consideration. This can be seen, for instance, in the work assessing how responses on variants of the three mountains task are influenced when the child is asked to turn away [7] or when the model scene is covered over during the test phase [8]. Even though this work is placed within the context of “perspective”, it clearly involves a memory component. The shielding manipulation also requires a child to consider the viewpoint of an agent who cannot see the display.

Irrespective of any difficulties, perspective-taking is often assumed to result from an *attribution process* in which humans *infer* what another sees (e.g., see [9], for a review). During the early 2000s, inference of visual perspective was incorporated into the related area of gaze following, the effect in which targets are processed more rapidly when located in a position looked at by another person [10]. The perspective-taking account of gaze following was tested with a series of experiments in which the agent’s ability to see was manipulated (e.g., [11]). The rationale here was that if the gaze following effect occurs because observers attribute *seeing* to the agent then no such effect should occur when an agent cannot see (at least at the looked-towards location). The agent’s ability to see was manipulated in a number of ways. For example, in one paradigm, the gazing agent would sometimes wear opaque goggles [12], whilst in another paradigm a physical barrier was sometimes placed between the agent and the critical object (i.e., target; [13]). There is still uncertainty and debate as to whether the attribution and inference of seeing does indeed modulate gaze following. The important point, however, is not whether this occurs, but that a number of researchers had already claimed that attribution and inference of another person’s viewpoint (i.e., their “perspective”) could drive social attention, and do so without the observer being asked to consciously consider what an agent may think or know (i.e., the effect occurs spontaneously). It is for this reason that the change in emphasis from attribution of vision to representation of visual experience, that occurred a decade ago, has to be seen as a significant development in perspective-taking thought.

## 3. Representation Rather than Function

Interest in adult perspective-taking was piqued in 2010 by the claim that that one particular form of perspective-taking (i.e., knowing that an agent can see a stimulus) can occur spontaneously [14]. The claim came from a paradigm in which observers are required to make a judgement about the number of dots located on the walls of a room. An agent (‘avatar’) is positioned in the centre and, by virtue of where they are looking, can either see the same number of dots as the participant or, on other trials, sees a different number. Typical results show that participants are quicker to discriminate dot number when the agent sees the same number, as opposed to when the agent sees a different number. The assumption has been that participants spontaneously represent what the agent can see. This leads to facilitated responses when she sees the same as the participant (with respect to the critical stimuli). A number of related procedures have now been developed all intended to examine perspective-taking. For example, in the *ambiguous number paradigm* [15,16], a single digit located in a scene may appear different for the participant and agent. Thus, the number “6” placed flat on a table would be seen as “9” when viewed by an agent sitting opposite. If a participant responds “9” when asked “what number is on the table”, they are said to have spontaneously taken the alternative perspective [17]. 

The Samson et al. paper initiated an abundance of work primarily centred on the issue of whether an agent’s perspective can be represented spontaneously. A related question is whether an observer can represent another person’s *visual experience*, spontaneous or not. By mentioning visual experience Samson et al., perhaps inadvertently, had highlighted the issue of representation, that is, addressing what it means to take another’s visual perspective. Current thinking was recently summarised by Ward, Ganis, and Bach [18], who stated, “A recent proposal is that VPT takes a (quasi-)perceptual form, “painting” a mental image of the content of another person’s viewpoint onto one’s perceptual system that can stand in for one’s own perception”. This was reiterated when the authors added that perspective-taking is akin to seeing an object “as if one would perceive it oneself”. Similarly, Moll and Kadipasaoglu [19] refer to perspective as being like a “snapshot” of a scene “in a literal, i.e., optical sense of the term”. The idea of visual experience being represented was also reiterated by Capozzi, Cavallo, Furlanetto, and Becchio [20] who stated that, “in simple perspective-taking tasks, one’s own and others’ visual experience influence each other”.

Such descriptions of perspective-taking should be seen as a very welcome development, seeking to address the issue of what it means to take another’s viewpoint. At the same time however, the above authors have not made clear what is meant by the notion that visual perspective-taking is, for example, “(quasi-)perceptual” and that humans can represent another’s snapshot perspective in a “literal” sense. One problem for the idea that visual perspective-taking is akin to perceptual experience is that it relies on the representation being able to code information pertaining to all aspects of the alternative viewpoint. As we set out below, a theory of visual perspective-taking needs to determine what exactly *can* be represented. We will also suggest that the language used to describe perspective-taking processes is critical. Thus, authors cannot *literally* mean that an observer can assume a perspective in a “literal” sense. Indeed, a milder approach to the idea that perspective-taking is (quasi-)perceptual can be adopted. However, without clarity it is difficult to know in what way this is meant. 

In sum, the past decade has seen a shift in emphasis in how perspective-taking is conceived. From attribution and inference, representation of another’s viewpoint is now said to be “quasi-perceptual” in that it is based on what another person experiences in a “literal sense”. We argue that this shift is to be welcomed because it begins to address the issue of what perspective-taking might actually mean. Indeed, it is this shift that necessitates a theory of *visual* perspective-taking. With the exception of issues concerning mental rotation and object recognition, prior to Samson et al. [14], “perspective-taking” was more associated with socially oriented processes rather than vision. Thus, it was the preserve of, for instance, developmental psychologists; vision scientists have not until recently examined “perspective-taking”. It is therefore not surprising that there is currently no theory concerned with what another person can actually see. Whatever the reason, clarity is required—a theory of perspective-taking is now needed.

Our position is of course predicated upon there currently being no such theory. Although perspective-taking has been subject to a vast amount of research and plays a major role is many theories (e.g., [21]), these are not theories of perspective-taking. When we say perspective-taking theory we mean a theory that is directly and only concerned with the act of representing (spontaneously or not) what another agent can see. It is interesting to note that Google searches (UK, December 2019) for “theory of attention”, “theory of memory”, “theory of emotion”, “theory of motivation”, and “theory of intelligence”, produce 16.5 million, 13.5 million, 7.5 million, 7.3 million, and 7.4 million hits respectively. If we make searches concern more specific psychological phenomena, we find that, for instance, “theory of motor control”, “theory of visual working memory”, “theory of episodic memory” and “theory of perception and action” produce 372 k (i.e., thousand), 78.4 k, 151 k, and 375 k hits respectively. Most significantly, “theory of social perspective-taking” produces 22.2 k hits. Compare all these with “theory of visual perspective-taking” which produces one single hit—a reference to the Qureshi, Samson and Apperly [22] *Cognition* article. This paper did not however present a theory of perspective-taking, nor was it intended to.

The central aim of the present article is to set out a number of issues and principles that a theory of perspective-taking might consider. Such a theory would not have to take into account all nine suggestions we make, or even the majority. Rather, they could provide a framework that shows what a theory could be concerned with and what it might mean to take another agent’s perspective. In our conception of perspective-taking, the process concerns what another person sees rather than what they know. Of course, an alternative theory could emphasise “perspective-taking” as the process in which *beliefs* are computed, with little consideration to what an agent can see. Thus, when we state that a theory should emphasise what another person sees (Point 1), this is only the case if the theory is concerned with *visual* perspective taking. Some of our suggestions may also be seen as problems with how perspective-taking is currently conceived. 

Building upon Cole and Millett [23], we first begin with an overarching suggestion; that the mental imagery debate provides a useful guide as to why a theory of perspective-taking is required, as was a theory of imagery. That debate also highlights some of the important issues that should be considered with any theory of visual perspective-taking. For example, a major problem that afflicted one side of the imagery debate (i.e., homunculus requirement) is also an issue for the notion of perspective-taking.

Fundamentally, both mental imagery and visual perspective-taking are concerned with representing a visual array that is not physically present, or at least in the case of the latter, an array that is different to the one seen by the observer. After virtually no work on the subject during the behaviourist-dominated years, the late 1960s saw the beginning of a huge amount of research on what was often called “the great debate” (e.g., [24]). This began when research moved from assessing how mental images aid learning and memory (e.g., [25]), to the nature of the images themselves and in particular how they are represented, i.e., the underlying cognitive structures. Amongst the various sub-issues, such as the degree to which the visual system is involved [26], the critical question concerned whether mental imagery is based on a pictorial representation. This, as we will see, is also central to the issue of visual perspective-taking. The argument, led by Kosslyn and colleagues (e.g., [27]), posited that images have a spatial structure, a structure that can aid the reasoning process. This was based on data from the central imagery paradigm. Results showed that the time it took observers to move their ‘mind’s eye’ over their image was directly related to the distance traversed. Images were thus said to have a spatial structure just like a real percept. This in turn suggested that observers could ‘look over’ and ‘read off’ information present in an image. It was therefore said to be the *image itself* that *caused* a response. The non-pictorial theory in contrast, led by Pylyshyn and colleagues (e.g., [28,29]), argued that images are represented symbolically. This position suggested that images, although real enough, do not aid the reasoning process. In this view, results from imagery experiments were said to occur because observers *know* what happens when viewing a real scene, e.g., it takes longer to traverse relatively larger distances. Observers then use this knowledge to (implicitly) simulate what would happen if they were looking at a real image. In this account, images do not help observers to generate responses; they are an effect not a cause. Put simply, the pictorial account argued that images occur in what might be called a ‘bottom-up’ manner in that they can be inspected. The non-pictorial, or symbolic, account in contrast argued that images are wholly generated by the observer; therefore, they cannot possess additional information that the observer does not already know.

Let us imagine that the mental imagery debate was solely concerned with the interesting empirical data that came from the vast number of experiments that were undertaken. In other words, the issue was not about the underlying mental representation but instead about phenomenology, such as whether there is a linear relationship between response time and distance traversed, whether the mind’s eye has a spatial resolution, when and what kind of images help with memory retrieval, and how experimental instructions modulate responses when participants ‘inspect’ their images. Had this been the situation, the Pylyshyn–Kosslyn discussion would not have had the prominence that it did; it would not have been elevated to the status of a “great debate”. The reason it was so important is that it concerned a central endeavour of cognitive science; the nature of underlying cognitive structures and the notion of representation. As Kosslyn, Pinker, Smith, and Shwartz [30] stated, “A history of mental imagery would almost require a complete history of the idea of mental representation, so intimate is the relationship between the two concepts”. This state of affairs in our history-of-science thought-experiment is equivalent to what we have today in the field of perspective-taking, i.e., a discipline dominated by assessing behavioural characteristics, with little consideration being given to representation. We argue that the field will benefit from addressing questions that examine mental representation. The specific ways in which the mental imagery debate can inform perspective-taking is provided throughout the following sections, particularly the next two.

### 3.1. A Theory of Visual Perspective-Taking Should Be Concerned with Vision

One aspect of the perspective-taking literature is its reference to and interchangeable use of the two basic forms of perspective-taking. This has its basis in the two very different meanings of ‘perspective’ and similarly ‘perception’. To the perceptual psychologist (e.g., a psychophysicist) these refer to the mechanisms and processes associated with constructing a ‘picture’ of the world. This would include, amongst many other processes, computation of luminance contrasts, edge detection, depth, and light wavelength subtractions. In contrast, for the more socially oriented psychologist they refer to the mechanisms involved with the processing of social information. Thus, one can ask about our ‘perception’ of the current prime minister or whether a person’s basic, political ‘perspective’ can be changed. Clearly, these are not concerned with edge detection and how a visual scene is constructed. Yet despite these clear differences, they are often discussed together. This is not surprising since both are concerned with attributing mental states to others. For example, the influential Apperly and Butterfill [21] article is both about visual perspective-taking as well as belief reasoning. To be clear, researchers can of course investigate, combine, and name any phenomena in any way they want. Our point concerns clarity in what any future theory is concerned with.

A theory of visual perspective-taking should therefore be unambiguously concerned with what another person can see. This necessarily means that if, for example, a ‘perspective-taking’ effect is not abolished when the agent’s view of the critical stimuli is blocked, then that paradigm is not concerned with visual perspective-taking (see [31,32,33]; note, as these references attest, the attempt to abolish a “perspective-taking” effect by blocking the line-of-sight has produced mixed/conflicting results). The same is true if an effect is not abolished when an agent is replaced by a non-agent, such as a chair (e.g., [34,35]). Such paradigms will therefore be of limited value in helping to inform or develop a theory of visual perspective-taking. If not due to vision, an effect may be due to an agent’s *knowledge* of what is in a scene rather than what they actually see. This is important for a theory of visual perspective-taking if only to make clear what the theory should not be about. Indeed, what is often attributed to a visual perspective may actually result from what an agent knows. For example, the basic effect in the dot-perspective paradigm might be due to the observer knowing that the avatar can only see, for example, one dot, which then interferes with the observer’s own view, in which two dots are seen.

**Figure 2:**
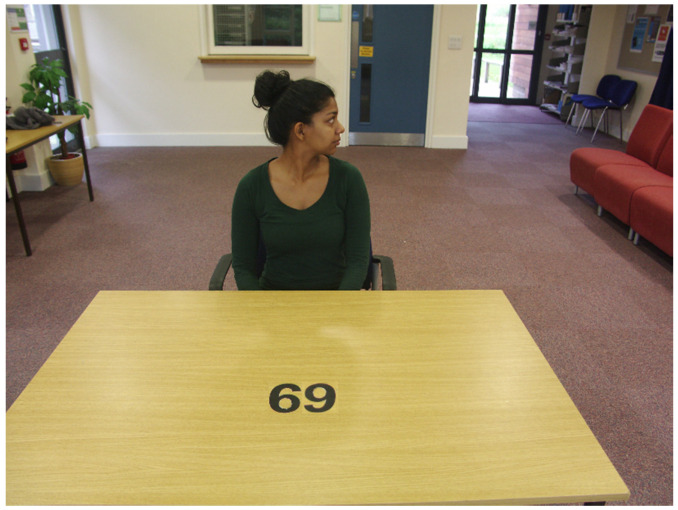
Since the agent is not looking at the critical stimulus, any effect caused by her presence cannot be due to the computation of her “perspective”.

This may seem somewhat odd given that the interference effect must in some sense result from an observer representing what the agent knows about the scene. However, vision and knowledge-of-vision are distinctly different as illustrated with the mental imagery debate. Recall that a central aspect of the debate was the question of whether paradigm results were due to what participants “saw” in their mental image (i.e., a vision phenomenon) or what they knew about how the world works (i.e., a knowledge phenomenon). To illustrate the important difference between vision and knowledge with respect to perspective-taking, consider Figure 2 (taken from Kuhn, et al., 2018). The agent is not looking at the number on the table. Our representation of what she is looking at, her visual perspective, cannot therefore include the number. Despite this, the agent/image still induces an interference effect when a participant is required to rapidly discriminate an ambiguous number such as 68 (seen as 89 from the agent’s position) compared with an unambiguous number such as 69 [16]; what some authors would call a “perspective-taking” effect. Indeed, Zhao, et al. [17] stated that participants had been “perspective-taking” in a variant of this paradigm in which observers made a non-speeded response to the question “what number is on the table?” As with the Kuhn et al. [16] experiment, this occurred in a condition where the agent was not looking at the digit. 

The reason for these effects is likely to be that the observer simply *knows* what the agent knows; she must have seen the number as she sat down. It is knowledge driving the phenomenon rather than vision. To reiterate, the spontaneous perspective-taking notion argues that it is an agent’s visual perspective, i.e., what they actually see, that is represented and it is this representation that interferes with the own observer’s perspective. It is, presumably, for this very reason that visual perspective-taking is said to be quasi-perceptual in which an agent’s perspective is computed in a literal optical sense. 

### 3.2. What Should a Model of Perspective-Taking Do?

A central aim of a perspective-taking model should be to specify the *type of content* that can and cannot be accessed by an observer when they consciously (i.e., non-spontaneously) attempt to adopt an alternative perspective. In other words, what features or properties that would be seen at the alternative position can reliably be “reconstructed”? One starting point for any perspective-taking model is the description given by Ward et al. [18]. Recall that these authors summarised the current view of perspective-taking by stating that it takes a “(quasi-)perceptual” form. At the very least, this has to mean that the relative position of objects is coded. This we know to be the case. For instance, Tversky and Hard [36] showed that observers can effortlessly represent where objects are in relation to each other with respect to an agent sitting opposite them. If perspective-taking is indeed quasi-perceptual this should additionally mean that relative metric distances between objects (or any stimuli points) are coded. If this is the case then observers should take progressively longer to judge attributes of two objects, seen from an agent’s viewpoint, as distance between the objects increases. It is also a reasonable assumption that perspective-taking mechanisms code for relative size; experience tells us that objects appear progressively smaller the further we are to them. For this reason, depth may also be a property that can be “resolved” when we perspective-take. 

One might say that when perspective-taking observers perform a *correction* to account for how a feature changes in accordance with the different position. These adjustments can presumably occur for certain object properties but not others. Thus, whereas relative location, including depth, can be resolved [36], apparent contrast is not likely to be corrected, and colours do not become more saturated as viewing distance is decreased. The basic paradigm could require participants to perform a judgement on a number of features and asked to do this from the perspective of an agent. This would allow the experimenter to systematically assess what can and cannot correctly be reconstructed from an alternative position. Although there will be some features of a scene that can be reliably resolved during perspective-taking, there will be other properties that cannot. For example, if asked to determine the relative spatial location of objects (e.g., an agent and monitor), observers are likely to perform at ceiling, as shown by Tversky and Hard [36]. This would contrast their ability to determine the colours seen. This is partly because the perception of colour can depend on viewing position. The present Figure 1 is another illustration of how difficult it can be to represent what colour is seen by an agent. It is however also true that if an agent is seeing a red object located on a table, an observer sitting opposite can be confident of the colour seen. It is an empirical question as to how reliable an observer will be when judging the colour seen by an agent. As we suggested above, an observer’s own experience of the visual world could also be included in a theory; although we cannot be certain of what colour another person is seeing, experience may provide a reliable indicator. One way to measure the accuracy with which we take others’ perspectives of colour is to present two participants with an object that one views through a colour filter; both participants then indicate on a colour scale what colour the other agent sees, and these two judgments are compared to determine success (see [37], for a task that requires little adaptation to meet these criteria). As well as stating what *can* be represented by an observer, any model of perspective-taking, therefore, needs to state what cannot. 

### 3.3. In What Sense Is a Perspective ‘Taken’ from an Agent?

Any model of perspective-taking should stipulate the sense in which information is ‘provided’ by the agent. The fact that ‘perspective-taking’ occurs on tasks where an alternative viewpoint is not relevant is often interpreted as evidence that other agents’ perspectives are generated spontaneously (e.g., [14]). Regardless of one’s standpoint on such an interpretation, if the whole notion of spontaneous perspective-taking is to mean anything, it has to mean that a perception from an alternative position, not immediately apparent, is then ‘given’ to the observer. Indeed, this is why the phenomenon is called perspective-*taking*; an observer acquires something (i.e., a viewpoint) they did not have before.

This is of course a matter of definition, and we clearly get *something* that we did not already possess, and this does not necessarily mean that we are provided with a complete and accurate representation of another’s viewpoint. For instance, an agent can provide us with access to information or interpretations we may not have previously considered. This can be seen in the *ambiguous number paradigm*; the agent effectively informs us that a “6” can also be seen as a “9”; the agent has given us something. Even here however, it is not clear *what* is being taken from the agent. Whilst it is tempting to say their “viewpoint”, or what they can see (i.e., their “perspective”) has been taken, we now know that inanimate objects without a perspective can create the same ambiguous number effect as a human agent does. For example, if the agent is replaced by a chair, the number “68” is as ambiguous as when the agent is there [35]. The chair effectively informs the observer that the number can also be “89”. We would not of course say that we have “taken the chair’s perspective”. It might turn out therefore that an agent simply provides us with a cue, a cue that suggests an alternative viewpoint. It is of course useful to call this a “perspective” but this would erroneously suggest that a *visual* perspective has been computed. The question is this section therefore is whether an observer takes something from an agent that is based on what is seen.

This is where one problem of perspective-taking resembles the central problem that afflicted one side of the mental imagery debate in an important way. Recall that the pictorial theory argued that images assist in the reasoning process; the information present in their representation can be used to solve appropriate tasks. This generated the notion that images can be “looked at” in order to glean information that was not available before. As opponents however often pointed out, this is a slightly awkward position to take because images are generated by the individual in the first place. Heil [38] provided an example to illustrate the problem. He instructs us to imagine (ex-) U.S. president Jimmy Carter and then asks us “how do you know it is Carter and not his twin or someone else disguised as Carter?” The implication here is that if we were challenged we might look a bit harder and proclaim “Oh yes, I think you’re right, it’s not him. I could have sworn it was! Are you sure it isn’t?” This bizarre interaction illustrates the problem as Heil and others saw it; one cannot generate an image of a stimulus without knowing what that stimulus is. It follows therefore that an image cannot give the observer something they did not have before.

This problem also exists for the notion that we can take another’s perspective because it is the observer themselves who is generating the alternative viewpoint. The issue of an observer already seeing/knowing something is inherent in all work on spontaneous perspective-taking. In the typical paradigm, the participant and the agent both look at the same stimulus, albeit from a different angle. The agent therefore sees the same thing as the observer in the consistent conditions, e.g., two red dots hanging on a wall. From this, it is not clear the degree to which responses are based on what the agent sees or what the participant sees. There are, however, a number of ways to present a physical stimulus to both an observer and agent that are perceived very differently by each. The present Figure 1 (after Pylyshyn, [28,29]) and the ambiguous images of Slezak [39] are both examples. In the latter, a stimulus is perceived as a particular animal only when viewed from a certain angle (i.e., 90 from upright). This angle could be that of the agent who is lying down; the animal would effectively be invisible to the participant. One could then examine whether this animal primes or influences responses on a range of tasks. If this is shown to be the case, then it would demonstrate that there is indeed something about an agent’s perspective that modulates responses—that their perspective was *taken*. Note that this experimental scenario is crucially different to other perspective-taking paradigms that employ ambiguous stimuli. In the ambiguous number procedure ([15]; see above), the dual-identity of the critical item (e.g., “6” or “9”) is known to the participant; in the procedure described above the stimulus seen by the agent is not seen and thus known to the observer.

The issue comes down to this; a perspective has either been generated solely by the observer themselves or it has occurred via the agent. This may seem odd. How can such information *not* be generated by the observer, instead generated “via the agent”? Indeed. If perspective information is generated solely by the observer, then, by definition, the observer has not *taken* a perspective; the agent will not have provided the observer with anything other than a cue that can influence responses. To put another way, the alternative viewpoint would not have given the observer anything that they did not know (implicitly) before. If this is the case then an agent’s viewpoint would not have served a function, it would be, dare we say, “epiphenomenal”.

A theory of perspective-taking has to therefore stipulate the mechanism or medium that can process a scene from an alternative location to that of the participant. This was a requirement of Kosslyn’s theory of mental imagery in which an *interpretive* mechanism equivalent to an “inner eye” was said to process an image (“the image is used”). Kosslyn, et al. [30] did describe such a mechanism that was developed as part of their mental imagery theory. It was this that they argued enabled information to be obtained from the image that was not available before the image was generated. The pictorial theory of mental imagery was however always plagued by the criticism that this mechanism is equivalent to a homunculus that can “see”. In the case of perspective-taking, it is a homunculus that needs to construct a scene from the alternative position. This requires access to the agent’s sensory system. The problem was demonstrated with the filters example shown at the beginning of our article. 

### 3.4. Attention and Perspective-Taking

**Figure 3:**
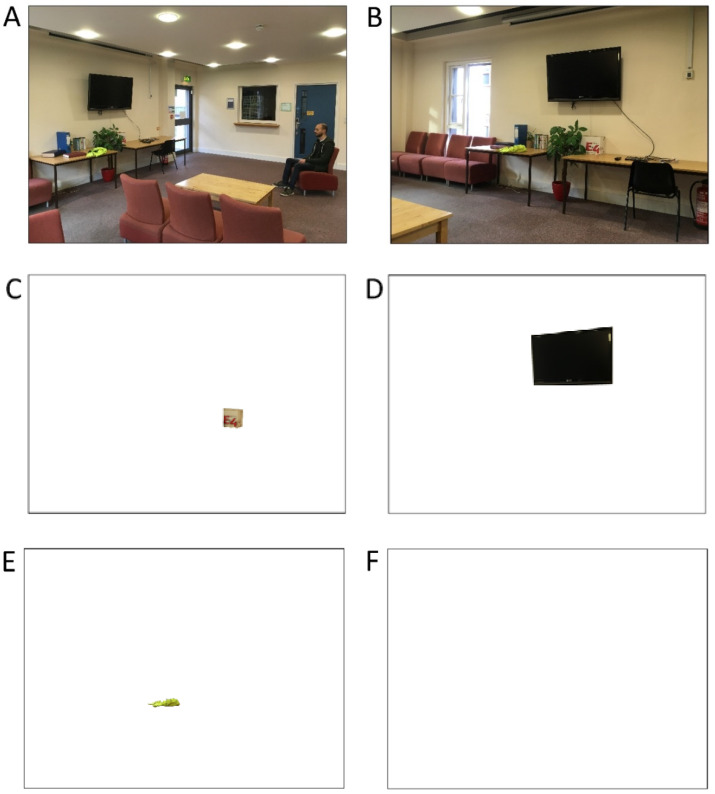
Attention and perspective-taking (**A**–**F**).

If asked to take the perspective of the person shown in Figure 3A, one might think that the scene would look similar to that shown in Figure 3B. This would be a reasonable assumption because the image in Figure 3B is captured by a camera placed at the same location (and facing the same direction) as the agent’s eyes in Figure 3A. Despite Figure 3B being a faithful representation of the scene, this was not the agent’s perspective. The large number of change blindness experiments revealed that our experience of a display-wide detailed visual representation of the scene in front of us is an illusion (e.g., [40,41]). If our attention is not directed at a location or object, we do not experience it. This explains why large changes to features of a display will go unnoticed unless attention is located at that position. It is the shifting of our attentional spotlight around a scene, which generates the illusion that all aspects of the array were represented at a relatively high level before our spotlight arrived. The perspective of the agent in Figure 3A therefore is likely to be closer to Figure 3C, or Figure 3D, or Figure 3E, etc. Their perspective may even be something more like Figure 3F, i.e., ‘zoning out’. Similarly, when you take the perspective of the agent your own representation of what he can see is also spotlight-like, but there is no good reason, at least for most scenes, why your spotlight and an agent’s spotlight will necessarily match up. This problem is magnified when we are viewing an agent who is located within a very busy real-world environment. There are multitudes of objects that could be currently attended.

Because we cannot reliably know where another person is attending we cannot be sure of what they are seeing, i.e., experiencing. This is even the case when we observe an agent looking directly at an object. For instance, if we see another person looking at a television screen we may not reliably know which aspect of the scene is being attended to by the agent. They may not even be attending to the television at all. In common perspective-taking paradigms such as the dot-perspective task there is virtually no ambiguity as to what the agent is looking at but as soon as a handful of objects are present and within their line-of-sight this will not be the case. Recall that perspective-taking is said to concern Theory of Mind, i.e., the mental state of others [42], and, as some authors suggest, it is the representation of a visual experience. Although one study has invoked the notion of change blindness and attentional allocation, in order to empirically motivate an experiment [43], the present authors cannot find any reference to the problem that attention creates for the notion that humans can take another’s perspective. A theory of perspective-taking must therefore say something about how another’s attention will influence the computation of a perspective. A theory might want to predict the *likelihood* that another agent is attending to a stimulus, given their gaze direction. This relates to the question of how informative a person’s gaze tends to be.

The issue of attention is where a theory of visual perspective-taking needs to draw upon Theory of Mind. Although our conception of a perspective-taking theory is very much concerned with vision, an attribution-of-thought component will however be necessary. This aspect could be informed by knowledge already uncovered by attention researchers. For instance, what an observer attends to is not solely due to their own top-down control (see [44,45]). The vast work on attentional capture has uncovered the stimulus properties that are particularly effective at modulating attention in a bottom-up way (see [46], for an early review). This work may be useful in bridging the gap between the internal process of attention, Theory of Mind, and visual perspective-taking.

### 3.5. How Informative Is Another Person’s Gaze?

Related to the question of agent attention is the issue of how reliable another’s gaze is in informing an observer of their visual perspective and mental state in general. Perspective-taking is after all concerned with others’ cognition; specifically what an agent sees. Visual cognition researchers have often assessed how effective eye gaze is in shifting an observer’s attention relative to other stimuli that have an informative directional component (e.g., [47]). A reasonable assumption has always been that gaze should be particularly effective because other individuals are conscious agents with a mind and what they look at might be important to an observer. This is often placed within a Darwinian context in which humans have been selected for their ability to read other minds. Work on gaze cueing has provided support for this assumption. For example, Wiese, Wykowska, Zwickel, and Muller [48] found that when observers believed an agent has intention, larger attention shifts occurred compared to when observers did not have this belief (see also, [49,50]). It is also the case, however, that a face exhibiting fear does not reliably induce greater cueing effects relative to a neutral face [16,51,52]. Furthermore, arrows seem to orient attention as well, if not better, than gaze [53,54,55]. This is surprising given the presumed importance of gaze for mental state attribution. We suggest a possibility that is rarely entertained in the gaze cueing and perspective-taking literature; gaze direction is informative on only a very small proportion of the time we spend viewing another person’s gaze. Consider the many occasions in which an observer interacts with other people or can simply see another human’s eyes. How often is their gaze informative? This is an empirical question, which could be addressed by recording an agent’s gaze during all their social interactions on a given day. The experimenter, or independent observers, could then view the footage and attempt to determine when a saccade is likely to be important for the person whom the agent is interacting with. Alternatively, one might consider the likelihood that eye gaze is indicative of subsequent behaviour. Eye gaze may turn out to be less informative that is often thought. Perhaps most importantly for perspective-taking theorists is the question of what information a person’s gaze conveys: is it *how* they are seeing something (i.e., Level 2 perspective-taking; [56]) or is it simply that they can see the item (i.e., Level 1 perspective-taking)? The latter is clearly very important in a large range of scenarios including efficient social interaction. However, knowing how they see something is not. This is the case for ‘turn-taking’ during conversation with a partner: their gaze direction is clearly important and informative but how they see us or anything else is not. The issue of how useful and informative gaze is when we look at another person is an empirical question and one that a theory of perspective-taking may want to address. Determining how often and, perhaps more significantly, the situations in which gaze *is* particularly important would provide a major contribution to any theory of perspective-taking.

### 3.6. Can We Take Our Own Perspective?

In order to adequately represent what another person can see, one has to be clear about what a perspective actually is. It is therefore useful to ask what it means to take *our own* perspective. When attempting to determine the precise nature of a phenomenon and what it means, researchers will often refer to its definition in commonly used dictionaries (e.g., Inhibition; [57]). The Oxford English, and Cambridge English, dictionaries provide definitions of perspective as an artist would conceive it. The Cambridge version states that a (visual) perspective is “the way that objects appear smaller when they are further away and the way parallel lines appear to meet each other at a point in the distance”. This description makes clear reference to perspective as a pictorial representation. In other words, a one-to-one mapping between what is represented and the representation itself. Indeed, when one attempts to draw a scene, it can help to consider each point as an x, y co-ordinate located in two-dimensional space; faithfully reproducing these will result in the generation of depth and size. Notice that this is the same as the Kosslyn’s notion of mental imagery; recall that an image was said to be based on a pictorial representation that had a linear spatial structure.

**Figure 4:**
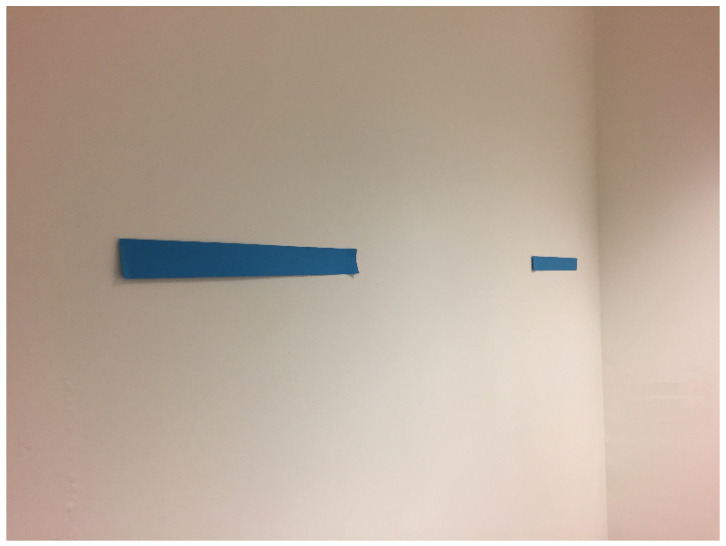
View of the stimuli as actually seen by participants.

Humans do not however seem to be particularly adept at taking their own perspective, as defined. Samuel, Eacott, and Cole [58] asked participants to stand 39 cm from a wall and look directly at two lines, both 32 cm in length, and offset to their right. Their view is shown in Figure 4. (Note that the photograph has not distorted this view; the figure faithfully represents the apparent size when viewed on a camera in the same position). 

It was emphasised that although the lines are identical in length, they should make a judgement on their apparent size. That is, their appearance rather than literal size, which participants knew to be the same. We found that 53% of participants stated that both lines looked to be the same length. This is striking given what they actually saw, as shown in the Figure.

It is, however, in hindsight, not surprising that participants were unable to represent the large difference in apparent size; visual experience, despite being partly derived from x, y co-ordinates, results from ‘later’ processes. It follows therefore that even if we can faithfully represent another’s perspective in terms of encoding relative spatial position (i.e., x, y co-ordinates) we will still not be able to represent what they can see; their perspective. A theory of perspective-taking might therefore tackle the issue of how the representation on which an alternative perspective is based relates to what the agent sees or experiences. 

### 3.7. What Does an Agent Provide?

**Figure 5:**
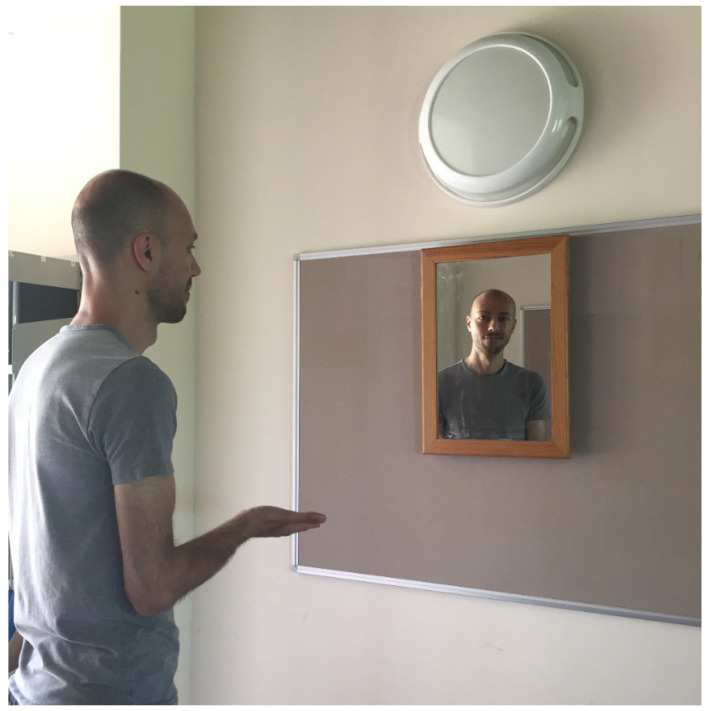
The Venus effect.

When a human agent is present in a display, responses on a variety of tasks are facilitated (e.g., [59]). It is not yet clear what exactly the agent provides that a non-agent does not. It may well turn out to be their perspective but, as noted above, there are a number of experiments showing that “perspective” effects are not abolished when the agent cannot see the critical stimuli (e.g., [13]). A model of perspective-taking might therefore want to suggest what exactly a human agent provides. If it is not computation of their visual experience, what might it be? Consider Figure 5: if asked to take the perspective of the man looking into the mirror and whether he can see his right hand as well as his face, people rarely give the correct answer. The correct answer is that he can see neither, he cannot see himself at all. This is referred to as the *Venus effect*, a familiar phenomenon often utilised during television and film production. If the agent/actor could see himself, the viewer would see the camera. (Note that a scene *can* be contrived such that the agent/actor sees himself but the observer does not see the camera). This is often taken as a good example of how poor humans are at reasoning about mirror reflections. It also shows how ‘perspective-taking’, far from being effortless, can be extremely limited. If it was effortless, the Venus effect would not exist at all. 

Interestingly, the Venus effect is significantly increased if the agent is replaced by, for instance, a camera [59]. In other words, reasoning about reflectance angles is better when observers are asked to take the position of another human as opposed to a non-human entity. This human-induced effect has been shown to occur in a variety of tasks. For example, adults are faster to judge what another person believes than what a note describes or a photograph depicts, even when they carry the same information [37,60]. It is currently not clear what another human provides that non-humans do not. It could be something as simple as an increase in overall reasoning ability; a type of social facilitation effect that can occur in the presence of another agent. One can imagine a problem solving experiment in which the solution cannot rely on a visual perspective (e.g., mathematic calculation), but the presence of an agent still increases performance. Only empirical investigation will reveal what exactly another agent provides in paradigms where human-present facilitation effects occur.

### 3.8. Language and Terms Are Critical

This penultimate point refers to how a model should *describe* perspective-taking, rather than the process of perspective-taking itself. There is a long history of how language is used in science to describe phenomena, particularly in the context of science communication and the lay public. As pointed out by Thomas Kuhn (e.g., [61]) there are inherent dangers when using metaphors to describe phenomena. The central problem is that metaphors can mistakenly be assumed to represent a phenomenon literally (see [62], for review) with one of the most contentious being the term “gene”. Opponents argue that the word suggests that genes literally exist, in the sense that strings of DNA could be separated into discrete entities. 

Recall that we welcome the recent comments concerning perspective-taking (e.g., [18]) because they attempt to describe what it might mean to take another person’s viewpoint. We do, however, urge caution and indeed reject any notion that perspective-taking involves the representation of another person’s “visual experience” or a “snapshot” in a “literal” sense. As we have stated, this would require a homunculus that has access to a person’s sensory system. Language is particularly important when describing perspective-taking because in some sense the observer is indeed representing another person’s visual experience, their perception, and we can of course represent many aspects of what another person can see (e.g., [36]). Metaphors do have their place in any scientific discourse as long as we are clear what they refer to, rather than solely relying on the metaphor to describe the phenomenon. Thus, one could imagine a perspective-taking theory arguing that the representation of an alternative viewpoint can be likened to the generation of a “picture in a literal sense” as long as this is qualified with a description of what this means. An example could be, “the coding of an agent’s viewpoint includes information pertaining to each point of a scene spatially relative to all other points in a one-to-one linear manner. The representation is akin to a three dimensional line drawing, or wire frame, but one in which surface reflectance properties are not reliably coded (e.g., colour)”. It is interesting to note that Kosslyn et al. [30] were criticised by Keenan and Olson [63] for using the term “literal” to describe the appearance of an object in a mental image without explaining what this meant. Rather than reply that this criticism was a straw man because this could not have meant that an image can literally exist in the brain, they instead set out what was actually meant by images existing in a “literal” sense. 

### 3.9. A Perspective-Taking Hypothesis

The central aim of the present article has been to highlight the need for a perspective-taking theory and what such a theory might want to consider. We have not suggested a theory ourselves. In this final section, however, we will posit a description of what might be the central process that occurs when an observer says that they are taking another person’s perspective. 

We start from the assumption that an observer rarely, if ever, needs to know how another person sees an object. In other words, “Level 2” perspective-taking is unnecessary. One might of course sometimes consciously consider how an item is seen from an alternative viewpoint but, given the level of computation involved (e.g., mental rotation), we suggest that this form of meta-cognition does not occur effortlessly or spontaneously (but see [18,43]). When we “perspective-take” the critical information we take from an agent is their gaze direction. Thus, mechanisms associated with gaze cueing signal what they are likely to be looking at. This itself is based on a given probability that the gaze is informative in the sense that the looked-at item is being attended. As we suggested in Section 3.4 above, this probability is yet to be empirically determined. Samson et al. [14] did suggest that gaze cueing mechanisms could play a mediating role in so-called spontaneous perspective-taking. We however argue that gaze following is *central* in assuming an “alternative view”. Once the system represents what the agent is looking at, via gaze following, our own perceptual mechanisms then represents the object (i.e., we look at it).

**Figure 6:**
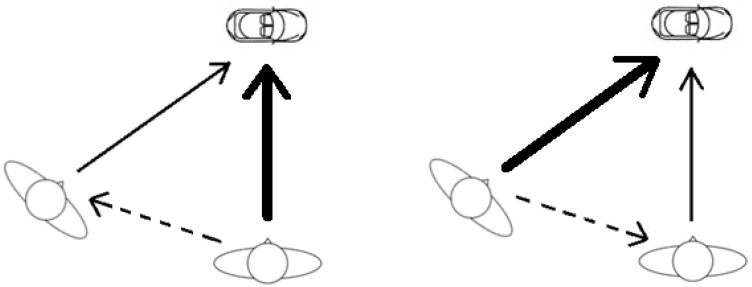
The relationships between agent, model, and stimulus.

The left panel of Figure 6 illustrates the current conception of perspective-taking. This however is an interpretation of the position; as stated, the precise description of what occurs in perspective-taking has not been presented. The information from an object is represented by the agent standing on the right (i.e., the emboldened line). What they see is then represented by the observer (standing on the left) who *takes* something from the agent, via Theory of Mind mechanisms (i.e., the dashed line). The observer clearly sees the object but this is deemphasised (hence, the thin line towards the stimulus). An important ‘direction of travel’ is from the agent to the observer. As we have argued, this relies too heavily on a homunculus to represent what the agent sees. We suggest that something more akin to the right panel occurs. The information from an object is again represented both by the agent and observer. The important difference in our conception however is that during perspective-taking there is no de-emphasis in what the observer sees, although as we state immediately below this is an empirical question. The critical feature derived from the agent is their gaze direction, as illustrated by the dashed arrow. What is referred to as “perspective-taking” is actually the representation of an item/stimulus from our own perspective; a perspective that has been induced by what another is looking at. One does also have to remember that, despite its name, perspective-taking does not seem to involve the generation of an alternative perspective, i.e., a different perception. One prediction that follows from the current conception of perspective-taking is that when an observer attempts to take an agent’s view their own perspective, although unchanged in any major way, is still somehow compromised. Given the vision and cognitive penetrability debate (see [64,65]), this could simply be due to increased attention toward the agent or a compromised ability to make judgments upon our perspective when perspective-taking, rather than compromised perception itself. Our alternative conception in contrast would predict a relatively smaller change in how an observer’s vision/attention is affected by perspective-taking. This would appear to be borne out by a recent study which found that taking other perspectives compromises subsequent judgments about the egocentric point of view (i.e., leads to a judgment erroneously based on the alternative perspective), but only very, very rarely [66]).

A major component of perspective-taking may, therefore, turn out to be no more than a type of “joint attention” [67] associated with gaze following. It cannot however be the only process. As we stated in Section 3.2, a model of perspective-taking needs to describe what can be reliably “resolved” at the alternative position when observers consciously attempt to adopt another’s perspective. Thus, although a perception does not change, an observer will still be able to reliably represent certain features from the agent’s position. As we argued, some of these features will have already been determined from the perspective-taking literature, e.g., relative object location [36].

## 4. Conclusions

We have argued that the perspective-taking notion needs reconsideration. As it currently stands the idea of representing what another person can see is vague and not well conceived. Rather than solely undertaking more empirical work assessing the circumstances under which perspective-taking occurs, authors should consider the type of representations that generate responses. We have therefore argued that a theory of perspective-taking is required and suggested the type of issues and questions it could address. 

The issues we have raised and points suggested pertain to a theory of *visual* perspective taking. In other words, representing what another person can see. This contrasts other forms of “perspective-taking” such as when an observer computes what an agent believes or knows. Central to our conception is the issue of what exactly can be taken from another person. What do they provide? We have suggested that some stimulus properties will be reliably computed, such as relative spatial location. Others, however, are less likely to be, for example, colour. Relatedly, we raise the question of whether an agent can provide an observer with information that they did not possess before. Although our conception of perspective-taking is concerned with vision, Theory of Mind processes are also important. An observer will need to know how likely an agent is attending to a stimulus. In essence, a theory of visual perspective-taking needs to stipulate the precise relationship between observer, agent, and object. We have also suggested that the debate surrounding mental imagery is very relevant to the perspective-taking notion, both in terms of problems (e.g., the homunculus requirement) and also in terms of questions raised. Indeed, at the risk of invoking ‘resemblance’ (see [68]), there are many similarities between the two phenomena, even at the level of terminology. For example, Newcombe [9] makes reference to the “mind’s eye” during perspective taking. 

We hope that recent and welcome references to what perspective-taking might actually entail (e.g., [18]) can motivate a fully developed theory of a phenomenon Piaget first described over two generations ago.
